# National Beef Quality Audit-2022 Phase 1: face-to-face and digital interviews

**DOI:** 10.1093/tas/txae034

**Published:** 2024-03-12

**Authors:** Colton L Smith, Tyler W Thompson, Keayla Harr, Macey Goretska, Thachary R Mayer, Trent E Schwartz, Sydni E Borders, Kerri B Gehring, Phil D Bass, Morgan M Pfeiffer, Gretchen G Mafi, Dustin L Pendell, J Brad Morgan, Davey B Griffin, Jeffrey W Savell, John A Scanga, Mahesh N Nair, Keith E Belk

**Affiliations:** Department of Animal Sciences, Colorado State University, Fort Collins, CO, USA; Department of Animal Sciences, Colorado State University, Fort Collins, CO, USA; Department of Animal and Food Sciences, Oklahoma State University, Stillwater, OK, USA; Department of Animal and Food Sciences, Oklahoma State University, Stillwater, OK, USA; Department of Animal Science, Texas A&M University, College Station, TX, USA; Department of Animal Science, Texas A&M University, College Station, TX, USA; Department of Animal Science, Texas A&M University, College Station, TX, USA; Department of Animal Science, Texas A&M University, College Station, TX, USA; Department of Animal Sciences, University of Idaho, Moscow, ID, USA; Department of Animal and Food Sciences, Oklahoma State University, Stillwater, OK, USA; Department of Animal and Food Sciences, Oklahoma State University, Stillwater, OK, USA; Department of Agricultural Economics, Kansas State University, Manhattan, KS, USA; iQFoods Co., Fayetteville, AR, USA; Department of Animal Science, Texas A&M University, College Station, TX, USA; Department of Animal Science, Texas A&M University, College Station, TX, USA; Department of Animal Sciences, Colorado State University, Fort Collins, CO, USA; Department of Animal Sciences, Colorado State University, Fort Collins, CO, USA; Department of Animal Sciences, Colorado State University, Fort Collins, CO, USA

## Abstract

The National Beef Quality Audit (**NBQA**) has been conducted regularly since 1991 to assess and benchmark quality in the U.S. beef industry, with the most recent iteration conducted in 2022. The goal of NBQA Phase I is to evaluate what needs to be managed to improve beef quality and demand. Interviews (*n* = 130) of industry personnel were conducted with the aid of routing software. In total, packers (*n* = 24), retailers (*n* = 20), further processors (*n* = 26), foodservice (*n* = 18), and allied government agencies and trade organizations (*n* = 42) were interviewed. Interviews were routed in software based on interviewee involvement in either the fed steer and heifer market cow and bull sectors, or both. Interviews were structured to elicit random responses in the order of determining “must-have” criteria (quality factors that are required to make a purchase), best/worst ranking (of quality factors based on importance), how interviewees defined quality terms, a strength, weakness, opportunities, threats (**SWOT**) analysis, general beef industry questions, and sustainability goals (the latter four being open-ended). Quality factors were 1) visual characteristics, 2) cattle genetics, 3) food safety, 4) eating satisfaction, 5) animal well-being, 6) weight and size, and 7) lean, fat, and bone. Best/worst analysis revealed that “food safety” was the most (*P* < 0.05) important factor in beef purchasing decisions for all market sectors and frequently was described as “everything” and “a way of business.” Culture surrounding food safety changed compared to previous NBQAs with interviewees no longer considering food safety as a purchasing criterion, but rather as a market expectation. The SWOT analysis indicated that “eating quality of U.S. beef” was the greatest strength, and cited that educating both consumers and producers on beef production would benefit the industry. Irrespective of whether companies’ products were fed or market cow/bull beef, respondents said that they believed “environmental concerns” were among the major threats to the industry. Perceived image of the beef industry in the market sectors has improved since NBQA-2016 for both fed cattle and market cow/bull beef.

## Teaser

The interviews portion of the National Beef Quality Audit revealed that food safety has become mandatory in the market, while cattle genetics and eating satisfaction were closely related for many. The beef industry was viewed favorably among the market segments, with suggestions for improvement including producer education and new marketing opportunities.

## Introduction

The National Beef Quality Audit (**NBQA**) was created within the framework of W. Edwards Deming’s concept of quality management (Smith, 1991; Petersen, 1999) that proved successful across many industries following WWII. Deming placed emphasis on self-assessment, benchmarking, and continuous improvement within a management scheme to increase productivity and quality of products while simultaneously reducing costs of production—thereby improving profitability and consumer demand for products (Petersen, 1999). In the late 1980s, in an effort to address the continued deterioration of consumer demand for beef, several industry leaders recognized that the beef industry lacked a comprehensive total quality management scheme; they initiated a goal to create one (Boleman et al., 1998). As part of this effort, it was necessary to determine where, within the beef supply chain, improvements could be made and controls could be exerted that would improve demand for and increase the quality of U.S. beef. Imperative to this vision was a need to focus on wholesale beef customers who made cattle and beef purchasing decisions: such as packers, further processors, retailers, and foodservice operators, to determine where perceived quality shortfalls occurred. Data gathered via the NBQA has been used to inform cattle producers of notable improvements and needs in quality and to help inform beef industry policy formation. Ultimately, the goal of the NBQA is to measure characteristics of importance for beef demand and to ascertain where controls are needed to reduce costs of production and improve profitability.

The United States beef industry has witnessed increased carcass weights, improved beef quality grades, and increased numbers of beef marketing programs (e.g., USDA-certified programs; Boykin et al., 2017; Hasty et al., 2017). Further, tenderness has improved since 1990, with a vast majority of beef now being considered “tender” or “very tender” (Gonzalez and Phelps, 2018). These accomplishments are partly due to the implementation of total quality management concepts in the beef industry and they showcase the success of the NBQA that allows the industry to “measure what they wish to manage.”

Domestically produced beef is comprised of about 80% fed market steers and heifers, while the remaining 20% is produced from market cows and bulls (USDA ERS—Livestock and Meat Domestic Data, 2023a). Therefore, investigators also conducted the National Market Cow and Bull Beef Quality Audit (**NMCBBQA**) jointly in conjunction with the NBQA for the second time, and both are considered under the umbrella of the NBQA-2022. Before the NBQA-2016, the two studies were not conducted simultaneously and data collection was solely focused on the fed steer and heifer market. The NMCBBQA was developed separately to gain similar knowledge about the market cow and bull beef market, which consisted of cattle that have been culled from their original purpose and sent to slaughter.

In 2022, the objective of the NBQA was to benchmark the beef industry’s quality shortfalls and image within the market sectors and government/trade organizations with ties to the beef supply chain, as well as to determine the relative importance of quality factors, assess where improvements have been made, and where future efforts could be made to continue to improve beef quality.

## Materials and Methods

Approval was attained before this study to work with human subjects through the Colorado State University IRB # 2106. Since no animals were used in this study, no Institutional Animal Use approvals were needed. Interviews were conducted with personnel representing U.S. beef market segments that make purchasing decisions concerning beef. This included personnel in packing companies, further processors, foodservice, and retailers. Additionally, representatives of peripherally aligned government and trade organizations (**GTOs**) involved with regulating and advocating for the beef industry also were interviewed. In total, 130 interviews were conducted through mixed modalities that included face-to-face, telephone, and video conference calls (necessary in 2022 due to the COVID-19 pandemic).

Individuals interviewed were heavily involved in purchasing beef products either as live animals, carcasses, wholesale cuts, case-ready retail products, and/or trimmings or other beef for further processing. Interviewees had knowledge of their companies’ purchasing criteria or were direct buyers of beef products. Like the 2016 Phase 1 National Beef Quality Audit (NBQA-2016), both market steer and heifer (fed beef) and market cow and bull beef interviews were combined into one study. Interviewees would designate at the start of the interview which sector they operated in and whether they purchased products derived from fed steers and heifers, market cows and bulls, or both. All interviews were conducted from July 2021 to November 2022.

### Computer-Assisted Interview Routing Software

A computer-assisted and routed interview sequence of questions was created using Qualtrics (Qualtrics 2021; Provo, UT, USA). Interviewers recorded answers based on lists of possible responses (or were added to possible responses at the time of the interview) and then the software routed to the next question based upon a selected answer. This thereby prevented questions being asked that were inappropriate for specific interviewees based on previous answers. Interviewers were responsible for asking questions and recording the provided answers within the software. When available, teams of two interviewers conducted each interview to ensure accuracy in reporting.

### Interview Overview

Routing and question sequencing allowed interviewees to provide instantaneous reactions without being led by questions to follow, thereby contributing to obtaining unbiased and accurate top-of-mind responses. The questions were asked in an order such that preceding questions would not affect answers to subsequent questions or provide information that might influence an interviewee’s perception of what the question was designed to ask. Thus, the interview resulted in gathering the interviewee’s initial thoughts and interpretation of the question.

Although predetermined quality factors (“categories” of quality interests) were selected by the investigators from which questions were asked, steps were taken to separate these quality factors from nonquality price factors. Therefore, the sequence of questioning was designed so that interviewees would not be thinking about economic criteria, but rather only the quality factors of interest that were germane to the objectives of the study.

To determine market coverage and direction of the interview, the interviews began with demographic questions. Answers to demographic questions routed the interview to the appropriate quality sections based on the market segment (i.e., packer, foodservice, retailer, further processor, or GTO) and market type (i.e., fed steer and heifer or cow and bull). The programmed software omitted financial questions for GTO, as those respondents did not purchase beef. If a company only participated in the fed steer and heifer market, they only answered questions from that perspective, and likewise for the market cow and bull companies/organizations. However, if they participated in both, they were able to answer separate but similar questions for each type of beef product. Each market segment respondent was allowed to list financial considerations (e.g., price—not an objective of the study) that determined their beef purchases and how much they would purchase. This allowed them to move from financial considerations to quality-related considerations as the interview proceeded.

Following the question about financial considerations in purchasing, an economic assessment of “must-have” quality factors was established. This was accomplished by asking in an open-ended format for respondents to list all traits that must be met to make a purchase. These traits were placed into one of the seven predetermined quality factors, which were 1) how and where cattle were raised, (2) lean, fat, and bone, (3) weight and size, (4) visual characteristics, (5) food safety, (6) eating satisfaction, and (7) cattle genetics. For a quality factor to confidently be a “must-have” characteristic, respondents must not have been willing to purchase an item lacking that trait for a discount. Thus, if they were willing to purchase an item for a discount that they previously listed as a “must-have” characteristic, it was no longer considered to be a “must-have” characteristic. Additionally, respondents were asked if they would be willing to pay a premium for a product that guarantees their “must-have” criteria.

To determine the relative importance of quality attributes via best/worst ranking, a series of eight questions were adapted from Igo et al. (2013) and Hasty et al. (2017) who used methods of Louviere and Islam (2008) and Cohen and Neira (2003). These were presented to interviewees from all market sectors and GTOs. In the first seven questions, three quality factors were given, and interviewees were asked to rank them in order of importance to their company, organization, or agency. The eighth question contained all seven quality factors and required that the respondents choose the most important and least important to their company, organization, or agency.

To understand the context of the best/worst rankings, respondents were asked to provide definitions for each of the quality terms that they reviewed. These questions were open-ended and phrased as, “What does [quality factor] mean to your company/organization?” All answers provided were recorded verbatim into the computer software.

Interviewee perceptions of the image, strengths, opportunities, weaknesses, potential threats, and changes since the last NBQA and NMCBBQA were explored next, followed by questions pertaining to sustainability. Each of these questions allowed open-ended responses. The final question asked interviewees to provide a synopsis of what positive and negative impacts the COVID-19 pandemic had on the beef industry. Entire responses were recorded verbatim into text boxes within survey software and were categorized into groups of similar responses for analysis. Results are reported as the frequency of similar responses per market sector.

### Statistical Analysis

Due to the fact that the NBQA and NMCBBQA are funded by the industry and conducted approximately every 5 yr since 1991, and because of the goal of monitoring trends in beef quality and demand metrics over time, analyses were performed similarly to those of Hasty et al. (2017) and Igo et al. (2013). Minor modifications were made to some of the analyses, and a brief overview of those modifications is provided here.

A general linear model with a binomial logit using the lmer4 package (version 1.1-31) in R (version 4.1.2) was used to estimate probabilities that a respondent would select a quality factor (i.e., a ‘category’) as a “must-have,” only if the quality factor was indeed a “must have”. This analysis was typical for an economic study.

Best/worst rank scaling was used to determine shares of preference for the quality factors, similar to that reported by Wolf (2013). Shares of preference were estimated using a multinomial logit model (Greene and Hensher, 2003) within SAS version 9.4 (SAS Institute Inc., Cary, NC). Estimated coefficients were then used to calculate shares of preference, similar to that reported by Lusk and Briggeman (2009). To assess differences in shares of preference among quality factors, a distribution of each estimated parameter was generated using a Monte Carlo procedure within Simetar (Richardson and Outlaw, 2008). In this application, probabilities generated a cardinal ranking system of relative importance. Mean separations of the calculated shares of preference were compared in R (version 4.1.2; R Core Team, 2022) using the base function ANOVA. This allowed for contrasts in magnitude difference between quality factors, meaning that if one share was two times greater than another, it is considered two times as important. Results associated with the fed steer and heifer markets were analyzed independently and are reported separately from those associated with the market cow and bull markets.

## Results and Discussion

### Demographics

**Figure 1.**
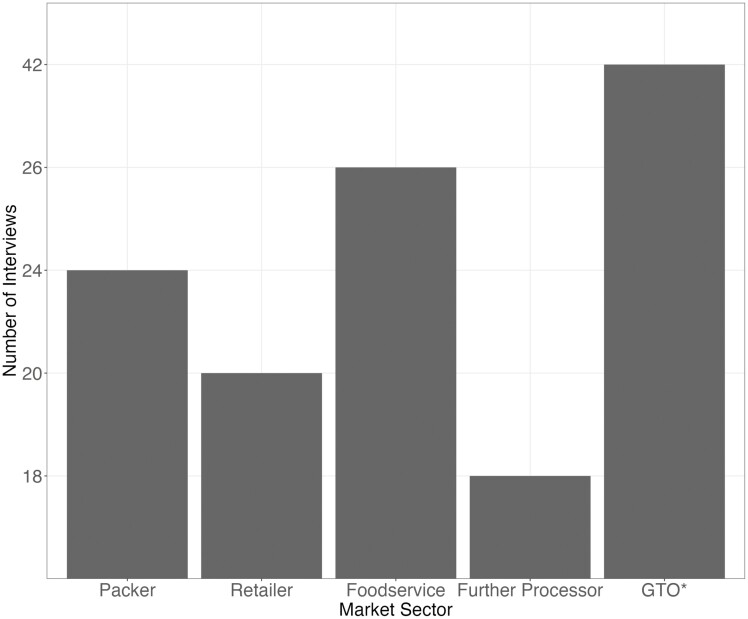
Number of interviews by market sector conducted for NBQA-2022 for both the fed steer and heifer and market cow and bull beef markets. *GTO = government or trade organization

Slightly fewer interviews were conducted during data collection for NBQA-2022 compared to the most recent NBQAs (Igo et al., 2013; Hasty et al., 2017) mainly because the study was conducted during the COVID-19 global pandemic and shortly thereafter. However, a substantial representation of the beef processing industry was maintained. The 24 packers interviewed represented a mixture of both the fed steer and heifer and market cow and bull sectors and represented greater than 90% of the total US slaughter capacity (Fig. 1). The packers consisted of companies that harvested between 15 and 30,000 head of beef cattle weekly. A total of 20 retailers were interviewed, constituting an estimated 40% to 45% of retail market share (Fig. 1; exact numbers remain unknown due to a lack of public information in this space). Retailers which included persons involved in beef procurement from companies that included small-scale butcher shops, big box and warehouse companies, and regional, national, and multinational grocers. Eighteen further processors participated in the interview process, while 26 interviews were conducted with the person(s) in the foodservice sector (Fig. 1). Further processors that were interviewed were diverse, and included smallbatch beef production through multinational companies. The coverage of the foodservice sector was greater than in previous audits, with an estimated 80% of the foodservice industry being represented (Hasty et al., 2017). Interviewees in the foodservice sector included fast-food, mid-scale casual dining, and foodservice distributors. Furthermore, 42 interviews were conducted with person(s) in government or trade organizations that are aligned or allied with U.S. beef production (Fig. 1).

In line with a trend discussed in Hasty et al. (2017), there was an increased appearance of dairy-type or dairy-crossbred animals being purchased, and further processors are more knowingly purchasing dairy and dairy-crossbreds to meet supply needs as the cattle cycle shifts to reduced total cattle numbers in the U.S. Moreover, all sectors reported purchasing imported beef both as live animals and as beef products (lean grinding beef); products from Australia, New Zealand, and Mexico comprised the bulk of imported beef. This finding corresponded with USDA data (USDA ERS, 2023b).

Compared to NBQA-2016, the number of branded beef programs and labels tied to production practices and credence attributes increased (Hasty et al., 2017). In 2016, packers reported sorting a mean of 7.6 branded beef programs per plant, while this value nearly doubled to an average of 14.5 per plant in 2022. Notably, the increase in the number of branded beef programs also translated into more programs at retail and foodservice establishments, but not at the further processor level of the industry. Retailers, foodservice operators, and further processors reported merchandising an average of 6.8, 6.9, and 2.3 branded beef programs, respectively, in 2022. Comparatively, in 2016, the same sectors reported the marketing 4, 2, and 7.3 branded beef programs per retailer, foodservice operator, and further processor, respectively (Hasty et al., 2017). A concern among the market sectors in NBQA-2016 was the lack of marketing programs; so, it is likely that the observed increase in branded beef programs identified in 2022 may have been a specific industry response to a need for beef product differentiation in the retail market space (Hasty et al., 2017).

A vast majority (83%) of interviewees reported that their companies require their suppliers to have live animal quality assurance programs in place. Live animal care programs are designed to promote animal well-being through providing industry and academically developed frameworks in animal handling, transport, and end-of-life care (Edwards-Callaway and Calvo-Lorenzo, 2020). Companies reported that they mostly rely on third-party auditing programs for verification of this requirement, including audits by those accredited by the Professional Animal Auditor Certification Organization (PAACO) and North American Meat Institute animal handling standards. Data also indicated that the national and state Beef Quality Assurance (BQA) programs, which now include animal handling and transportation programs implemented by the industry, were required by 28% of companies interviewed. Compared to Hasty et al. (2017), this was a sharp increase in the requirements for implementation of national and state BQA programs. Most likely, the increase in a number of required animal care programs stems from consumer concerns about animal well-being (Edwards-Callaway and Calvo-Lorenzo, 2020; Today’s Beef Consumer, 2022) and industry efforts to educate downstream market sectors about BQA.

### Packers

“Food safety” garnered the greatest (*P* < 0.05) share of preference (85.2%) and was clearly the most important quality factor for packers when contrasted with lean, fat and bone (2.2%), weight and size (2.4%), visual characteristics (1.5%), eating satisfaction (3.7%), how and where cattle are raised (1.4%), and cattle genetics (3.6%; Table 1). Similar to findings of Phase 1 in the last two NBQAs, packers reported most frequently that “food safety” is “everything” (69%) or “a way of business” (42%) for their companies (Table 2). But strikingly, many packers shared the sentiment that without rigorous food safety standards in place, their companies would not be able to conduct business and that downstream users of their beef products would find a different supplier. Thus, this suggested a robust culture shift and that “food safety” has become a pillar in their business structures. The likelihood of “food safety” being a must-have for packers decreased from 31% in 2016 to 10% in 2022 (Table 3; Hasty et al., 2017), and only two packers interviewed would pay a premium to ensure “food safety” in products they purchase (Table 4).

**Table 1.** Shares of preference^1^ (±SE) for the relative importance of quality factors for steer and heifer beef market.

Quality factor	Packer (*n* = 17)	Retailer (*n* = 16)	Foodservice (*n* = 25)	Further processor (*n* = 18)	GTO^2^ (*n* = 42)
Weight and size	2.4^c^ (1.3)^3^	2.8^c^ (0.9)	6.3^c^ (1.2)	4.0^e^ (1.0)	12.3^c^ (3.9)
Visual characteristics	1.5^c^ (0.8)	2.0^d^ (0.7)	3.8^f^ (0.8)	6.2^c^ (1.4)	10.1^d^ (3.3)
Cattle genetics	3.6^b^ (1.9)	12.0^b^ (3.5)	19.8^b^ (3.4)	20.2^b^ (4.4)	23.0^a^ (6.3)
Eating satisfaction	3.7^b^ (1.9)	10.9^b^ (3.2)	19.7^b^ (3.2)	20.1^b^ (4.1)	23.1^a^ (6.6)
How and where cattle were raised	1.4^c^ (0.8)	2.9^c^ (1.0)	5.4^d^ (1.0)	5.3^d^ (1.2)	7.4^e^ (2.6)
Lean, fat and bone	2.2^c^ (1.2)	2.7^c^ (0.8)	4.6^e^ (0.9)	6.4^c^ (1.5)	7.4^e^ (2.7)
Food safety	85.2^a^ (7.3)	67.8^a^ (8.3)	40.5^a^ (5.8)	37.9^a^ (7.0)	16.6^b^ (4.7)

^1^Shares of preference = probabilities in a cardinal ranking system of relative importance allowing for contrasts in magnitude difference between quality factors, meaning that if one share was two times greater than another, it is considered two times as important.

^2^Government or trade organization

^3^Standard error of the mean for the shares of preference within each market sector.

^a,b,c,d,e,f^Shares of preference without a common superscript within the same column are different (*P* < 0.05).

**Table 2.** Categorized responses from interviewed companies defining predetermined quality factors to their company/organization in relation to purchasing beef from both the fed steer and heifer market and the market cow/bull beef markets.

	Packer (*n* = 24)		Retailer (*n* = 20)		Foodservice (*n* = 18)		Further processor (*n* = 18)		GTO^1^ (*n* = 42)	
Quality factor	Most frequent^2^	Definition	Most frequent	Definition	Most frequent	Definition	Most frequent	Definition	Most frequent	Definition
Lean, fat and bone	57%	Yield	88%	Lean to fat ratio	58%	Lean to fat ratio	71%	Lean to fat ratio	57%	Lean to fat ratio
	38%	Lean to fat ratio	27%	Yield	35%	Yield	36%	Bone/structure	29%	Yield
	23%	Carcass weight and size	11%	Quality grade	23%	Beef x dairy	21%	Yield	21%	Quality grade
Weight and size	58%	Carcass weights	50%	Uniformity in cattle	35%	Uniformity in cattle	29%	Uniformity in cuts	48%	Carcass weights
	35%	Uniformity in cattle	39%	Uniformity in cuts	35%	Uniformity in cuts	21%	Uniformity in cattle	40%	Uniformity in cattle
	35%	Uniformity in cuts	33%	Carcass weight	35%	Carcass weights	21%	Carcass weight	24%	Uniformity in cuts
Visual characteristics	54%	Phenotypic attribute	39%	Marbling	38%	Appropriate product color	43%	Lean/trimmed product	69%	Phenotypic attributes
	23%	Marbling	33%	Appropriate product color	38%	No defects	36%	Phenotypic attributes	23%	Consumer appeal
	15%	Appropriate product color	22%	Consumer appeal	35%	Phenotypic attributes	36%	Appropriate product color	21%	No defects
	15%	Lean/trimmed product	22%	Phenotypic attribute			36%	Consumer appeal		
Food safety	69%	Everything	61%	Everything	65%	Everything	57%	Everything	64%	Everything
	42%	Way of business	39%	Way of business	23%	Products/materials produced in effective food safety environments	36%	Way of business	28%	Way of business
	11%	Foreign material elimination	22%	Products/materials produced in effective food safety environments	19%	Foreign material elimination	28%	Products/materials produced in effective food safety environments	28%	Products/materials produced in effective food safety environments
Eating satisfaction	69%	Customer satisfaction	67%	Customer satisfaction	85%	Customer satisfaction	71%	Customer satisfaction	83%	Customer satisfaction
	27%	Flavor	33%	Tenderness	46%	Overall palatability	43%	Overall palatability	26%	Overall palatability
	23%	Overall palatability	28%	Flavor	27%	Tenderness	14%	Tenderness	26%	Tenderness
					27%	Flavor			24%	Flavor
Cattle genetics	54%	Quality genetics/improving herd	55%	Quality genetics/improving herd	50%	Quality genetics/improving herd	28%	Quality genetics/improving herd	64%	Quality genetics/improving herd
	35%	Genetic potential for marbling	35%	Genetic potential for marbling	31%	Angus	14%	Beef type	36%	Genetic potential for marbling
	23%	Beef × dairy	28%	Beef type	15%	Beef type	14%	Genetic testing/genetic markers	33%	Genetic testing/genetic markers
							14%	Profitability		

^1^Government or trade organization

^2^Most frequent = top three most common responses and ties. Response data were evaluated as the number of times that interviewees in each market sector identified the attribute as a definition or description of the given category by the total number of responses for each market sector.

**Table 3.** The probability that a quality factor was considered a must-have factor for all beef purchases for the steer and heifer beef market.

Quality factor	Packer (*n* = 17)	Retailer (*n* = 16)	Foodservice (*n* = 25)	Further processor (*n* = 18)
How and where cattle were raised	0.13^1^	0.17	0.00	0.11
Lean, fat and bone	0.00	0.06	0.14	0.16
Weight and size	0.07	0.11	0.18	0.11
Visual characteristics	0.10	0.00	0.09	0.16
Food safety	0.10	0.39	0.13	0.47
Eating satisfaction	0.03	0.11	0.00	0.11
Cattle genetics	0.03	0.00	0.00	0.00
None	0.24	0.00	0.09	0.21

^1^Probabilites were determined by a binomial logit, and represent the likelihood that a quality factor was a “must-have” for a market sector.

**Table 4.** Number of companies interviewed that were willing to pay a premium for a guaranteed quality factor for both the fed steer and heifer and the market cow/bull beef markets.

Quality factor	Packer (*n* = 24)	Retailer (*n* = 20)	Foodservice (*n* = 26)	Further processor (*n* = 18)
Weight and size	2	0	7	1
Visual characteristics	3	1	0	0
Cattle genetics	2	3	1	0
Eating satisfaction	2	1	5	5
How and where cattle were raised	3	2	3	1
Lean, fat and bone	2	2	3	2
Food safety	2	2	8	3

Packers reported declining shares of preference for the “eating satisfaction” quality category compared to NBQA-2016 (Hasty et al., 2017). This was true even though packers suggested that “eating satisfaction” was important to their companies. Most packing sector respondents defined the “eating satisfaction” category as “having customer satisfaction,” “good flavor,” and “high overall palatability” at 69%, 27%, and 23%, respectively (Table 2). It could be that the emphasis on eating quality by the industry has reduced complaints from consumers in this category, and is no longer a primary concern to packers. Similar to food safety, most packers were not willing to pay a premium to ensure “eating satisfaction.” Interestingly, the “cattle genetics” quality category increased shares of preference compared to previous quality audits, with 54% of packers defining “cattle genetics” as “high-quality genetics that improve the herd,” which was often paired with “genetic potential for marbling,” and 23% of packers recognizing the utilization of dairy-crossbred cattle as a source for high eating quality characteristics (Igo et al., 2013; Hasty et al., 2017). Another potential reason that more emphasis has been placed on “cattle genetics” for packers could be the monetary premium that animals that qualify for branded programs provide (Schroeder et al. 2021).

“Weight and size,” “visual characteristics,” “how and where cattle were raised,” and “lean, fat, and bone” were least important (*P* < 0.05; Table 1) to packers as quality categories in the fed steer and heifer market. In comparison to NBQA-2016, “weight and size” shifted from “cattle size” (40% in 2016; Hasty et al., 2017) to predominantly “carcass weights” (58%) and “uniformity in cattle” and “uniformity in cuts” in 2022. For packers, this change was most likely due to pressures tied to meeting supply demands that were also meeting their customers’ specifications during the latter portion of the COVID-19 pandemic. Packers defined “visual characteristics” placing more emphasis on phenotypical traits such as hide color and marbling (Table 2). Although not conclusive, the association of phenotypic attributes, including hide color, muscling, and marbling is suggestive that packers are using visual characteristics to help predict the quality of the resulting carcasses.

Similar to results of NBQA-2016 and -2011, packers overwhelmingly defined “lean, fat, and bone” as “yield” (57% of responses), while “lean-to-fat ratio” was included as a definition for this quality category 38% of the time, and 23% said that it meant “carcass weight and size” to their company (Table 2; Igo et al., 2013; Hasty et al., 2017). Although the quality category of “how and where cattle are raised” gained more attention with other market segments, for packers in the fed steer and heifer markets, this was among the least important quality factors. For quality factors of “weight and size,” “visual characteristics,” “how and where cattle were raised,” and “lean, fat and bone,” packers were not likely to classify them as a “must-have” criteria before purchasing (Table 4), and few reported that they would pay a premium to guarantee one of these quality factors. In the current beef market, it is now common for cattle feeders and packers to have vertical-alliance contracts, which helps to create beef products that meet customer specifications (Schroeder et al., 2021). Due to the way these cattle are marketed between feeders and packers, it is likely that the packers interviewed were able to source cattle that met their companies’ goals (i.e., Certified Angus Beef, no beta-agonists, hormone-free, upper two-thirds choice, etc.) that were also at the expected weight and size.

Similar to the fed steer and heifer market, packers that participate in the market cow and bull beef sector placed the greatest importance on “food safety,” which received about three times (*P *< 0.05) the shares of preference (37.5%) compared to “weight and size,” “cattle genetics,” and “eating satisfaction”—each of which resulted in 12.4%, 12.5% and 12.4% of shares of preference, respectively (Table 5). For this market, packers put more emphasis (*P *< 0.05) on “lean, fat, and bone” and “how and where cattle were raised” compared to the fed market, and placed the least importance (*P* < 0.05) on “visual characteristics.” Data for the relative importance of quality factors in the market cow and bull beef sector can be observed in Table 5. The emphasis on “lean, fat, and bone” is not surprising for the market cow and bull beef sector because much of this product is used for further processing (such as grinding and value-added muscle cuts) and is not quality-graded compared with most beef products in the fed steer and heifer market.

**Table 5.** Shares of preference^1^ (±SE) for quality factors for the market cow and bull beef market.

Quality factor	Packer (*n* = 13)	Retailer (*n* = 5)	Foodservice (*n* = 8)	Further processor (*n* = 7)
Weight and size	12.40^b^ (2.4)^2^	2.10^e^ (2.0)	6.13^d^ (3.1)	5.67^d^ (1.9)
Visual characteristics	3.92^e^ (1.0)	0.41^f^ (0.5)	3.67^e^ (2.1)	7.21^c^ (2.3)
Cattle genetics	12.47^b^ (2.6)	12.58^b^ (12.1)	18.24^b^ (8.8)	16.31^b^ (5.3)
Eating satisfaction	12.41^b^ (2.4)	10.77^c^ (8.7)	17.93^b^ (8.0)	16.09^b^ (4.3)
How and where cattle were raised	9.47^d^ (2.0)	1.57^e^^,^^f^ (1.5)	2.21^f^ (1.4)	4.07^e^ (1.5)
Lean, fat and bone	11.60^c^ (2.2)	4.26^d^ (3.9)	7.28^c^ (3.9)	16.12^b^ (4.6)
Food safety	37.47^a^ (6.2)	68.33^a^ (20.3)	44.54^a^ (15.2)	34.52^a^ (8.9)

^1^Shares of preference = probabilities in a cardinal ranking system of relative importance allowing for contrasts in magnitude difference between quality factors, meaning that if one share was two times greater than another, it is considered two times as important.

^2^Standard error of the mean for the shares of preference within each market sector.

^a,b,c,d,e,f^Shares of preference within each column without a common superscript differ (*P *< 0.05).

### Retailers

The most important quality factor (*P* < 0.05) for retailers was “food safety” (67.8%; Table 1), which has not changed since the previous two NBQAs (Igo et al., 2013; Hasty et al., 2017). However, shares of preference allocated to “food safety” increased with retailers over the past decade, from 38.8% in 2011 to 68% in 2022. Further, retailers most frequently defined “food safety” as “everything” (61%), and “a way of business,” and “products/materials produced in effective food safety environments” with 39% and 22%, respectively, constituting the most frequent answers (Table 2). Thirty-nine percent of retailers reported that “food safety” was a “must have”, but again, few retailers were willing to pay a premium to guarantee that food safety was adequate—it was an expectation for doing business. Moreover, a greater number of retailers said that “food safety” was a “must have” compared to NBQA-2016. Increased emphasis on “food safety” for retailers may be due to the impacts of product recalls on retail companies. While the packer or further processor may ultimately be at fault for a food safety concern in manufactured beef products sold at retail, the retailer is directly involved with the consumer and may be seen as more responsible for the food safety risk compared to the manufacturer (Ni et al., 2014). So retailers’ requiring “food safety,” and even those willing to pay a premium to guarantee it, may be attempting to safeguard against recalls and the consequential decline in sales, or to prevent civil liability within an establishment.

The next most important quality factors for retailers were “cattle genetics” and “eating satisfaction,” garnering 12.0% and 10.9% shares of preference, respectively (Table 1). Compared to previous NBQAs, “cattle genetics” substantially increased in importance to retailers (Igo et al., 2013; Hasty et al., 2017). This was most likely due to marketing program influences (e.g., Certified Angus Beef and other breed-based programs) which retailers associated with greater “eating satisfaction.” “Cattle genetics” were most frequently defined by retailers as “quality genetics” (55%) and “genetic potential for marbling” (35%; Table 2). To better explain, one retailer said that “Improved genetics improve the end product.” A similar sentiment was expressed among several retailers. Moreover, two-thirds of retailers defined “eating satisfaction” as “customer satisfaction”, whereas 33% of interviewees defined it as tenderness and 28% included “flavor” in their response (Table 2). Due to retailers associating improved genetics with better eating satisfaction, and higher quality to more “customer satisfaction,” we found it reasonable to conclude that retailers are associating “cattle genetics” with “eating satisfaction.”

“Weight and size,” “visual characteristics,” “how and where cattle were raised,” and “lean, fat, and bone” were all similar (*P* > 0.05; Table 1) in relative shares of preference for retailers at 2.8%, 2.0%, 2.9%, and 2.7%, respectively. The definitions of these quality factors to retailers can be further examined in Table 2. Furthermore, the likelihood of “weight and size,” “visual characteristics,” “how and where cattle were raised,” and “lean, fat, and bone” to be a “must-have” purchasing criteria decreased since NBQA-2016 (Hasty et al., 2017).

Retailers involved in the market cow and bull beef market placed six times the importance on “food safety” compared to any other quality factor (*P* < 0.05; Table 5). In a similar pattern to the fed steer and heifer market, retailers placed “cattle genetics” and then “eating satisfaction” as the second and third (*P* < 0.05) most important quality factors. The relative importance of these traits was an increase from previously reported data (Hasty et al., 2017). “Lean, fat, and bone” was fourth in importance (*P *< 0.05) with 4.3% of shares of preference. “Weight and size,” “visual characteristics,” and “how and where cattle were raised” completed the rankings with two percent or fewer shares of preference (Table 5).

### Foodservice

For foodservice companies interviewed, “food safety” was the most important (*P* < 0.05; Table 1) quality factor at 40.5% of shares of preference. In fact, 65% of foodservice interviewees directly stated that “food safety” is “everything” to their companies. Another 23% defined it as “products are produced in an effective and food safe environment,” with 19% relaying the importance of “foreign material elimination.” Although other market sectors also mentioned foreign material contamination, the foodservice sector was most likely to describe the importance of it during their daily operations. Interestingly, “food safety” was not as likely to be a “must have” criterion for making a beef purchase compared to previous NBQAs (Hasty et al., 2017). Like other market sectors, most foodservice companies were not willing to pay a premium to guarantee “food safety” (Table 4), which indicated that foodservice companies expect “food safety” in products they purchase.

Like responses gathered from retailers, foodservice companies placed greater importance on both “cattle genetics” (19.8%) and “eating satisfaction” (19.7%) compared to previous NBQAs (Igo et al., 2013; Hasty et al., 2017). This increase in importance for “cattle genetics” was closely associated with “eating satisfaction” due to the foodservice companies linking eating quality experiences to cattle genetics. Further, foodservice operators often defined cattle genetics as “quality genetics that improves the cattle herds” and “Angus” at 50% and 31%, respectively. Results from NBQA-2016 showed that breed, and specifically the word “Angus,” appeared most in interviewee definitions of cattle genetics (Hasty et al., 2017). Although less frequent in the 2022 version, “Angus” was still of genetic importance among nearly 1/3 of foodservice companies interviewed. Overwhelmingly, 85% of foodservice provided “customer satisfaction” as the definition for “eating satisfaction.”

“Weight and size” received 6.3% of shares of preference from foodservice operators; more than any other market sector, excluding GTO (Table 1). The minor increase in the relative importance of this quality factor was most likely due to foodservices’ desire to standardize the weight and size of portioned cuts, along with grinding criteria (Committee, 2015). “Weight and size” were defined 35% of the time as “uniformity in cattle size”, “uniformity in cuts”, and “uniformity in carcass weights.” The importance of uniformity was summarized directly by one foodservice company: “We are interested in the ability to actually cut through product, and for it to be uniform.” Additionally, 18% of foodservice companies were more likely to require a product that was the desired weight and size. The emphasis placed on uniformity within this market sector is likely due to the struggles that the beef industry was facing at the time of the interviews. It was repeatedly mentioned among this market sector that workmanship and product specifications were not being met, leading to the lack of uniformity in the beef products they were purchasing from upstream supply chain vendors.

“How and where cattle were raised” received 5.4% shares of preference, “lean, fat, and bone” received 4.6% shares of preference, and “visual characteristics” rounded out the bottom with 3.8% shares of preference for foodservice companies (Table 1). Moreover, 58% of foodservice companies defined “lean, fat, and bone” as “lean to fat ratios” and placed importance on the yield of the beef products they purchased. “Visual characteristics” were frequently described in relation to “lean, fat, and bone” as foodservice companies suggested high proportions of fat were less desirable to their customers. However, when directly asked “what does visual characteristics mean to their company,” 38% of foodservice companies defined it as “appropriate product color” and “no defects.”

Foodservice companies rated “food safety” as the most important quality factor (*P* < 0.05) in purchasing decisions for the market cow and bull beef sector (Table 5). A total of 65.4% of foodservice companies reported purchasing market cow/bull ground beef and/or trimmings. Complete rankings for foodservice in the market cow and bull beef market are provided in Table 5.

### Further Processor

Similar to the other market sectors, “food safety” was the most important (*P* < 0.05) quality factor for further processors (Table 1). When asked, “What does food safety mean to your company?” 57% of further processors said “everything.” Further processors also defined “food safety” as a “way of business” (36%) with one further processor going as far as to say that “Unsafe product means bad business.” The likelihood of food safety being required for a further processor to purchase beef was 47%, which was numerically the highest of any of the supply chain market segments (Table 3).

The quality factors “cattle genetics” and “eating satisfaction” were of equal (*P* > 0.05; Table 1) relative importance to further processors. “Cattle genetics” were most frequently (28% of the time) defined as “quality genetics that improves the herd,” while “genetic testing/genetic markers,” and “beef type cattle” each appeared in 14% of responses. A number of further processors reported that they use genetic testing to guarantee the genetics of their beef and that has increased their profits (Table 2). The most common definition of “eating satisfaction” was “customer satisfaction” in 71% of responses for further processors, similar to findings in NBQA-2016 (Hasty et al., 2017). The meaning of “customer satisfaction” was better understood based on the following quote from a further processor, “[Eating satisfaction is] very important for people to be repeat customers.” However, foodservice companies were less likely to consider cattle genetics in purchasing decisions and most would not have paid a premium for it but would pay premiums for guaranteed eating satisfaction, underscoring the importance of eating satisfaction for this market sector (Tables 3 and 4).

“Lean, fat, and bone” and “visual characteristics” were the third most important (*P* < 0.05) quality factors for further processors at 6.4% and 6.2% shares of preference, respectively. Further processors defined “lean, fat, and bone” as “lean to fat ratio” 71% of the time and “bone/structure” 36% of the time, with another 21% of responses including the word “yield” (Table 2). The response tied to “bone” was frequently used in relation to the bone being present in the final product that should have been boneless. “Visual characteristics” were reported by further processors as meaning “lean/trimmed products” in 43% of responses; “phenotypic attributes (e.g., marbling, color, external fat, etc.),” “appropriate product color,” and “consumer appeal” were used in 36% of responses for this market sector. Of least importance (*P* < 0.05) to further processors was “how and where cattle were raised,” followed by “weight and size,” 5.3% and 4.0%, respectively (Table 1).

Further processors involved in the market cow and bull beef industry reported “food safety” to be numerically twice as important (*P* < 0.05) as any other quality factor. Differing from responses by interviewees in the other market sectors, this segment ranked “lean, fat, and bone,” “cattle genetics,” and “eating satisfaction” together with approximately 16% shares of preference each (Table 5). “Lean, fat, and bone” was most commonly defined as “lean to fat ratio,” and further processors noted that in the context of cow and bull beef products, it is primarily used as raw materials in their beef grinding and value-added products.

### Government or Trade Organizations

GTO respondents do not purchase beef cattle or beef products, but rather regulate and provide service to each of the market sectors evaluated. Therefore, they work closely with those who make beef purchasing decisions and thus are privy to vital information that may impact the U.S. beef supply system, along with issues that may evolve in the future for the industry. When interviewing GTO respondents, purchase criteria questions were omitted, but GTO respondents still participated in providing best/worst scaling, provided definitions for quality factors, and provided their inputs on the current state of the beef industry.

For GTO respondents, “cattle genetics” and “eating satisfaction” were ranked highest based on shares of preference (20.2% and 20.1%, respectively; *P *< 0.05; Table 1). To these organizations and agencies, “cattle genetics” meant that the U.S. beef herd has improved in quality over the past several years and that (in 36% of responses) genetics were related to the “potential for marbling.” Following the themes of responses in other market sectors, this finding related closely to “eating satisfaction.” “Eating satisfaction” was most often defined as “customer satisfaction” (Table 2). One GTO respondent said, “[Eating satisfaction] is major for our trading partners; they want the products and part of the ranch experience, which comes down to the eating experience.”

In previous NBQAs, “food safety” was the most important quality factor for GTO respondents, but in the present study, it was second in importance (*P* < 0.05), with only 16.6% of shares of preference (Table 1; Igo et al., 2013; Hasty et al., 2017). This perhaps represents progress in beef safety and documents that it is no longer a primary initial reaction to questions pertaining to beef quality. Interestingly, concerns previously mentioned in the NBQA-2016 (Hasty et al., 2017) regarding drug residues remaining in meat tissues were much less frequent and the industry was recognized for low prevalence of drug residues in beef in NBQA-2022, with one GTO respondent saying, “The beef industry does an exceptional job at having a low prevalence of antibiotic residues in meat.” Nonetheless, “food safety” was still important to GTO respondents, as they characterized this quality factor 64% of the time as “everything,” but also frequently mentioned how well the beef industry has historically addressed food safety issues.

“Weight and size” was next most important (*P* < 0.05) to GTO respondents and were defined as “carcass weights,” “uniformity in cattle,” and “uniformity in cuts” (percentages and data available in Tables 1 and 2). Following “weight and size” with about two percentage points fewer shares of preference, was “visual characteristics.” For GTO respondents, this quality factor related more to “phenotypic attributes,” which appeared in 69% of answers. Specifically, definitions contained answers including hide color, boxed beef program specifications, and eating quality traits. Rounding out the relative importance with 7.4% for GTO was “how and where cattle were raised” and “lean, fat, and bone.” The GTO interviewed were less concerned about these quality attributes, but admittingly claimed it is because these two things are not the most impactful for their duties as organizations/agencies.

## Image, Strengths, Weakness, Opportunities, Threats, and Changes from Previous Audits

### Image

The image of the beef industry from the perspective of interviewed companies, government organizations, and allied trade organizations is crucial to understanding how the beef industry is perceived by those making buying decisions in the differing market segments of the beef industry. To ascertain the image of the beef industry from interviewees, the question, “How does your company view the image of the fed steer/heifer or market cow/bull industry?” was asked as an open-ended question. Overall, the market sectors and allied organizations had a positive view of the fed steer and heifer market (Table 6). In fact, over half of the packers, foodservice, further processors, and GTO respondents claimed they viewed it “good” or “very good.” A few interviewees expressed that they believed their company had a negative image of the fed steer and heifer market. Most frequently, the negative image expressed by packers, further processors, and foodservice was related to an apparent contempt towards packer profit margins. Further, retailers had the most negative view of the fed steer and heifer market, and although for multifactorial reasons, the most common complaints concerned sustainability efforts and animal well-being. Worth noting as well, due to retailer’s position in the supply chain and their distance from cattle production, it is likely that they were less familiar with standard beef production practices. Nonetheless, the overall image of the fed steer and heifer market has improved over the past 5 yr along the beef supply chain and among GTOs interviewed.

**Table 6.** Categorized responses from interviewed companies explaining what they believed as the image of the fed steer and heifer beef market.

Packer (*n* = 17)		Retailer (*n* = 16)		Foodservice (*n* = 25)		Further processors (*n* = 18)		GTO^1^ (*n* = 42)	
Most frequent^2^	Response	Most frequent	Response	Most frequent	Response	Most frequent	Response	Most frequent	Response
29.4%	Very good	0.0%	Very good	24.0%	Very good	27.8%	Very good	26.1%	Very good
41.2%	Good	25.0%	Good	36.0%	Good	38.9%	Good	23.9%	Good
0.0%	Improving	25.0%	Improving	4.0%	Improving	0.0%	Improving	10.9%	Improving
0.0%	Reputable	12.5%	Reputable	4.0%	Reputable	5.6%	Reputable	15.2%	Reputable
23.5%	Needs improvement	6.3%	Needs improvement	24.0%	Needs improvement	16.7%	Needs improvement	0.0%	Needs improvement
0.0%	Bad	25.0%	Bad	0.0%	Bad	5.6%	Bad	4.3%	Bad

^1^Government or trade organization

^2^Most frequent = top three most common responses and ties. Response data were evaluated as the number of times that interviewees in each market sector identified the attribute as a definition or description of the given category by the total number of responses for each market sector.

In the market cow and bull beef market, the beef industry image has improved over the past 5 yr among the market sectors (Table 7). In NBQA-2016, respondents from all market sectors listed concerns about animal well-being and public perception of the nature of market cows and bulls (Hasty et al., 2017). In NBQA-2022, the perceived image of market cow and bull beef appeared to improve compared to NBQA-2016. Additionally, respondents across market sectors listed that they noticed improved animal well-being efforts compared with responses in NBQA-2016, which might have also contributed to the improved perceived image reported for market cow and bull beef (Hasty et al., 2017).

**Table 7.** Categorized responses from interviewed companies explaining what they believed as the image of the market cow and bull beef market.

Packer (*n* = 13)		Retailer (*n* = 5)		Foodservice (*n* = 8)		Further processor (*n* = 7)		GTO^1^ (*n* = 35)	
Most frequent^1^	Response	Most frequent	Response	Most frequent	Response	Most frequent	Response	Most frequent	Response
30.8%	Very good	60.0%	Very good	14.3%	Very good	12.5%	Very good	14.3%	Very good
15.4%	Good	20.0%	Good	20.0%	Good	37.5%	Good	20.0%	Good
15.4%	Improving	20.0%	Improving	0.0%	Improving	25.0%	Improving	0.0%	Improving
15.4%	Reputable	0.0%	Reputable	14.3%	Improving	0.0%	Reputable	34.3%	Reputable
15.4%	Needs improvement	0.0%	Needs improvement	0.0%	Needs improvement	0.0%	Needs improvement	0.0%	Needs improvement
0.0%	Unknown	0.0%	Unknown	42.8%	Unknown	0.0%	Unknown	0.0%	Unknown

^1^Government or trade organization

^2^Most frequent = top three most common responses and ties. Response data were evaluated as the number of times that interviewees in each market sector identified the attribute as a definition or description of the given category by the total number of responses for each market sector.

### Strengths

Overwhelmingly, respondents across all market sectors were quick to say that “product quality” is the greatest strength of the fed steer and heifer market (Table 8), the same finding reported by Hasty et al. (2017). “Product quality” was often referred to as “flavorful”, “tender” and “desired” among the interviewees. Furthermore, another common strength identified was “food safety,” and many respondents identified that the “lifestyle of the American cowboy” was a strength of beef production, and believed that the use of this imagery could be a strong marketing tactic for American beef. In the market cow and bull sector, two main strengths were identified. First, the “pricing of market cow and bull beef” was a main strength, providing high value at a lower price point (Table 9). However, it is likely that this strength will vary depending on cattle production cycles and on supply. The “secondary value” of market cow and bull beef was defined by one foodservice company as “It provides outlets for producers, and a low-quality product is transformed into a high-quality processed product that meets consumers’ standards.”

**Table 8.** Categorized responses from interviewed companies explaining what they believed to be the strengths of the steer and heifer beef market.

Packer (*n* = 17)		Retailer (*n* = 16)		Foodservice (*n* = 25)		Further processors (*n* = 18)		GTO^1^ (*n* = 42)	
Most frequent^2^	Response	Most frequent	Response	Most frequent	Response	Most frequent	Response	Most frequent	Response
47.1%	Product quality	43.6%	Marketing programs	44.0%	Food safety	38.9%	Consistency	60.7%	Product quality
41.2%	Food safety	31.3%	Taste	44.0%	Product quality	33.3%	Product quality	23.9%	Lifestyle
35.3%	Diversity of supply	18.8%	Product quality	32.0%	Taste	22.2%	Food safety	19.6%	Food safety
29.4%	Efficiency	18.8%	Consistency	24.0%	Availability	16.7%	Efficiency	15.7%	Consistency of supply
23.5%	Marketing programs	18.8%	Lifestyle			16.7%	Lifestyle		

^1^Government or trade organization

^2^Most frequent = top three most common responses and ties. Response data were evaluated as the number of times that interviewees in each market sector identified the attribute as a definition or description of the given category by the total number of responses for each market sector.

**Table 9.** Categorized responses from interviewed companies explaining the strengths of the market cow and bull beef market.

Packer (*n* = 13)		Retailer (*n* = 5)		Foodservice (*n* = 8)		Further processors (*n* = 7)		GTO^1^ (*n* = 35)	
Most frequent^2^	Response	Most frequent	Response	Most frequent	Response	Most frequent	Response	Most frequent	Response
38.5%	Consistency	40.0%	Food safety	60.0%	Reduced price	28.6%	Secondary value	34.5%	Secondary value
30.8%	Availability	40.0%	Reduced price	20.0%	Secondary value	14.3%	Product quality	23.3%	Availability
23.1%	Reduced price	20.0%	Product quality	20.0%	Availability	14.3%	Consistency	10.0%	Marketing
		20.0%	Secondary value	20.0%	Product quality	14.3%	Availability	10.0%	Reduced price
						14.3%	Reduced price		

^1^Government or trade organization

^2^Most frequent = top three most common responses and ties. Response data were evaluated as the number of times that interviewees in each market sector identified the attribute as a definition or description of the given category by the total number of responses for each market sector.

### Weaknesses

Weaknesses identified for the fed steer and heifer beef industry were heavily influenced by the COVID-19 pandemic. Many of the respondents identified that “labor shortages” were affecting their businesses (a weakness that is not rooted in the primary production of cattle). Additionally, “increased costs” and an “inconsistent supply” were identified as weaknesses by retailers, foodservice, and further processors (Table 10), which were heavily influenced by the conditions created by the pandemic. Moreover, fragmentation of the industry, and especially animosity towards beef packers, was prevalent among the market sectors and within GTO respondents’ remarks. There was clear animosity for the large beef packers by respondents, mostly due to perceived financial inequities within the supply chain that were thought to benefit large beef packers at the expense of other market sectors and smaller-scale packers.

**Table 10.** Categorized responses from interviewed companies explaining what they believed as the weaknesses of the steer and heifer beef market.

Packer (*n* = 17)		Retailer (*n* = 16)		Foodservice (*n* = 25)		Further processor (*n* = 18)		GTO^1^ (*n* = 42)	
Most frequent^2^	Response	Most frequent	Response	Most frequent	Response	Most frequent	Response	Most frequent	Response
29.4%	Lack of traceability	25.0%	Cost	36.0%	Supply	27.8%	Cost	30.4%	Too fragmented
23.5%	Packer animosity	25.0%	Lack of traceability	32.0%	Too fragmented	27.8%	Packer animosity	13.0%	Product quality
23.5%	Too fragmented	18.8%	Food Safety	24.0%	Cost	22.2%	Too fragmented	10.9%	Supply
11.8%	Lack of labor	18.8%	Supply	24.0%	Packer animosity				

^1^Government or trade organization

^2^Most frequent = top three most common responses and ties. Response data were evaluated as the number of times that interviewees in each market sector identified the attribute as a definition or description of the given category by the total number of responses for each market sector.

Market cow and bull beef were characterized by the identification of similar weaknesses to those identified for fed steer and heifer markets, along with several others (Table 11). First, there were major concerns about the “public perception” of the market cow/bull beef industry. Specifically, respondents were concerned about the condition of cattle at slaughter or that consumers may view cow and bull beef as  “old cattle” that are used for food and may view this practice negatively. This was often coupled with concerns about animal welfare, with common complaints that echoed findings from Hasty et al. (2017); timely culling, animal mobility, and foreign object contamination (e.g., “buckshot”) are impacting animal well-being. Perhaps increased animal well-being for the market cow and bull beef market could prove to be more financially lucrative for cattle producers as more timely and strategic culling procedures are implemented via BQA programming (Parsons and Stockton, 2022). Therefore, increased producer education regarding timely culling practices could prove to be valuable for cattle producers.

**Table 11.** Categorized responses from interviewed companies explaining weaknesses of the market cow and bull beef market.

Packer (*n* = 13)		Retailer (*n* = 5)		Foodservice (*n* = 8)		Further processor (*n* = 7)		GTO^1^ (*n* = 35)	
Most frequent^2^	Response	Most frequent	Response	Most frequent	Response	Most frequent	Response	Most frequent	Response
38.5%	Perception	50.0%	Perception	20.0%	Too fragmented	28.6%	Foreign objects	46.9%	Product quality
23.1%	Supply	25.0%	Supply	20.0%	Supply	28.6%	Supply	25.0%	Supply
15.4%	Too fragmented	25.0%	Poor marketing	20.0%	Product quality	28.6%	Product quality	18.8%	Animal well-being
15.4%	Foreign objects	25.0%	Too fragmented	20.0%	Perception	28.6%	Perception	15.6%	Food safety

^1^GTO = government or trade organization

^2^Most frequent = Top three most common responses and ties. Response data were evaluated as the number of times that interviewees in each market sector identified the attribute as a definition or description of the given category by the total number of responses for each market sector.

### Opportunities

In NBQA-2022, a new question was asked to determine where respondents believed that the fed steer and heifer industry had potential opportunities. Although the answers to this question were quite varied (Table 12), a few responses were re-occurring. First, respondents for which their supply chain market segment was closer to consumers were quick to identify that consumer psychographics are changing and new methods for marketing beef, such as e-commerce, could be advantageous to the industry. However, most major retailers already provide online commerce through websites and mobile phone applications. Moreover, these same respondents suggested that increasing the beef industry’s consumer education efforts could help to increase consumer awareness of beef production. Increased consumer education efforts are also desired by 72% of consumers who wish to know and understand modern food production (Consumer Demands for Food, 2022). Similarly, increasing beef producer education on best cattle production practices could help to increase beef quality. However, interviewees did not elaborate on which areas of producer education that they hoped to see. Opportunities for the market cow and bull beef were thematically similar to the fed beef market but focused heavily on increasing education for producers on animal well-being practices concerning timely culling and mobility, and potential means for reducing foreign material contamination (i.e., buck/birdshot; Table 13).

**Table 12.** Categorized responses from interviewed companies explaining for opportunities for the steer and heifer beef market.

Packer (*n* = 17)		Retailer (*n* = 16)		Foodservice (*n* = 25)		Further processor (*n* = 18)		GTO^1^ (*n* = 42)	
Most frequent^2^	Response	Most frequent	Response	Most frequent	Response	Most frequent	Response	Most frequent	Response
29.4%	Increase product quality	50.0%	Changing consumer	36.0%	Changing consumer	38.0%	Supply chain equitability	37.0%	Increase education initiatives
17.6%	Increase value added	31.0%	Increase education initiatives	16.0%	Niche markets	30.8%	Increase product quality	34.8%	Increase product quality
11.8%	Export markets	18.0%	Niche markets	16.0%	Increase education efforts	23.1%	Niche markets	21.7%	Export markets
11.8%	Changing consumer	12.3%	Animal disease	16.0%	Increase product quality	15.4%	Changing consumer	21.7%	Changing consumer
11.8%	Increase education initiatives								

^1^Government or trade organization

^2^Most frequent = top three most common responses and ties. Response data were evaluated as the number of times that interviewees in each market sector identified the attribute as a definition or description of the given category by the total number of responses for each market sector.

**Table 13.** Categorized responses from interviewed companies explaining opportunities for the market cow and bull beef market.

Packer (*n* = 13)		Retailer (*n* = 5)		Foodservice (*n* = 8)		Further processor (*n* = 7)		GTO^1^ (*n* = 35)	
Most frequent^2^	Response	Most frequent	Response	Most frequent	Response	Most frequent	Response	Most frequent	Response
58.3%	Producer education	60.0%	Consumer education	40.0%	Increase profits	33.3%	Increase profits	26.7%	Increase profits
33.3%	Consumer education	40.0%	Producer education	40.0%	Producer education	33.3%	Supply regulation	36.7%	Producer education
25.0%	Supply regulation	20.0%	Supply regulation	14.3%	Supply regulation	16.7%	Producer education	17.2%	Consumer education
8.3%	Foreign objects					16.7%	Foreign objects	14.3%	Traceability

^1^Government or trade organization

^2^Most frequent = top three most common responses and ties. Response data were evaluated as the number of times that interviewees in each market sector identified the attribute as a definition or description of the given category by the total number of responses for each market sector.

### Perceived Threats

“Environmental concerns” appeared to be the greatest perceived threat across the industry for the fed steer and heifer beef market (Table 14). Respondents worried about drought impacts, changing corporate sustainability initiatives, consumer demand for sustainable beef, and potential government regulation of beef production to be more “environmentally friendly.” Interestingly, environmental concerns are currently not at the forefront of consumers’ minds when making beef purchasing decisions, but rather animal well-being is the most major concern (Today’s Beef Consumer, 2022). So, environmental issues tend to be tied more to business initiatives than to current consumer perceptions.

**Table 14.** Categorized responses from interviewed companies explaining threats to the steer and heifer beef market.

Packer (*n* = 17)		Retailer (*n* = 16)		Foodservice (*n* = 25)		Further processor (*n* = 18)		GTO^1^ (*n* = 42)	
Most frequent^2^	Response	Most frequent	Response	Most frequent	Response	Most frequent	Response	Most frequent	Response
47.1%	Labor shortages	31.3%	Cyber security	36.0%	Environmental concerns	38.9%	Environmental concerns	33.3%	Environmental concerns
47.1%	Environmental concerns	25.0%	misleading labels	36.0%	Labor shortages	22.2%	Activist	28.3%	Public perceptions
35.3%	Activists	18.8%	Conglomeration of industry^3^	32.0%	Lack of consumer education	16.7%	Federal regulations	28.9%	Activists
29.4%	Animal disease	18.8%	Federal regulations	24.0%	Activists			22.2%	Animal disease

^1^Government or trade organization

^2^Most frequent = top three most common responses and ties. Response data were evaluated as the number of times that interviewees in each market sector identified the attribute as a definition or description of the given category by the total number of responses for each market sector.

^3^Conglomeration of industry = less market competition due to a lower number of companies and higher volumes being produced by few companies.

Interestingly in NBQA-2022, 31% of retailers perceived “cyber security” to be a major threat to beef production and their own operations (Table 14). In general, cyber security can be defined as “the protection of theft or damage to information technology hardware, software, and the data stored on the systems” (Huxtable and Schaefer, 2016). Similar to other crimes, a digital attack can result in access to information unlawfully, or lead to ransomware and other exploitation (Pandey et al., 2020). In a retailers position, a cyberattack, could reveal sensitive financial information, be used to disrupt supply chain management or be a gateway to upstream (distributors, further processors, packers) market segments information technology systems as the companies within a supply chain are technologically linked (Pandey et al., 2020). This could mean that vulnerabilities in cyber security at any segment of the beef supply chain could have impacts on another, and potentially disrupt the beef supply chain.

Respondents from differing supply chain market sectors had slightly different perceived views of threats regarding the market cow and bull beef industry. Notably, they were mostly concerned with public perception and the impacts that a biosecurity issue, such as Foot and Mouth Disease (FMD), could have on the industry (Table 15). Additionally, respondents across market sectors expressed concern about the lack of traceability in the beef industry to address animal health and export needs. Although there is a commercially available vaccine for FMD, it is not used currently in the U.S. because of trade ramifications for doing so (Diaz-San Segundo et al., 2017), and other diseases must be addressed on a case-by-case basis. A more robust traceability system would allow for better containment of such animal health issues if they occurred in the United States. Concern for lack of traceability was expected, as this was a frequently reported concern in NBQA-2016 (Hasty et al., 2017). Although the market segments and GTO expressed more desire for increased traceability within the beef supply chain, the beef industry has been reluctant to do so citing increased production costs on mainly cow–calf producers (Shear and Pendell, 2020). This is something that requires additional attention during the next 5 yr.

**Table 15.** Categorized responses from interviewed companies explaining threats to the market cow and bull beef market.

Packer (*n* = 13)		Retailer (*n* = 5)		Foodservice (*n* = 8)		Further processor (*n* = 7)		GTO^1^ (*n* = 35)	
Most frequent^2^	Response	Most frequent	Response	Most frequent	Response	Most frequent	Response	Most frequent	Response
53.8%	Public perception	40.0%	Public perceptions	60.0%	Public perception	57.1%	Animal disease	28.1%	Activists
38.5%	Alternative proteins	40.0%	Federal regulations	20.0%	Animal disease	28.6%	Activists	25.0%	Animal disease
15.4%	Activists	20.0%	Cost	20.0%	Regional Access	28.6%	Public perception	18.8%	Public perception
15.4%	Animal disease	20.0%	Animal disease			14.3%	Cost		
		20.0%	Alternative proteins			14.3%	Food Safety		

^1^Government or trade organization

^2^Most frequent = top three most common responses and ties. Response data were evaluated as the number of times that interviewees in each market sector identified the attribute as a definition or description of the given category by the total number of responses for each market sector.

### Impact of NBQA Phase I

To gauge the impact of the NBQA Phase 1, respondents were asked to describe any changes they have noticed since the previous NBQA was conducted. For the fed steer and heifer industry, most companies and GTO respondents interviewed were aware of previous NBQAs (Table 16). These companies said that the beef industry has “become more efficient” and that “nothing” has changed over the last 5 yr. The beef industry has become more efficient over the past 50 yr (Capper, 2011), however, the marked increase in efficiency identified in NBQA-2022 by respondents may also have been related to improvements in the supply chain during the COVID-19 pandemic. In the market cow and bull beef sector, fewer respondents were aware of the NBQA (or, conversely, the NMCBQA), but of those that were, animal well-being has improved or “nothing has changed” (Table 17). Reported increased animal well-being by the market sectors was an improvement from NBQA-2016 which indicated that animal well-being needed improvement in the market cow and bull beef sector, and suggested that efforts to improve animal handling at slaughter are becoming more effective (Hasty et al., 2017).

**Table 16.** Categorized responses from interviewed companies explaining what changes they have noticed since the 2016 NBQA for the fed steer and heifer beef markets.

Packer (*n* = 17)		Retailer (*n* = 16)		Foodservice (*n* = 25)		Further processor (*n* = 18)		GTO^1^ (*n* = 42)	
Most frequent^2^	Response	Most frequent	Response	Most frequent	Response	Most frequent	Response	Most frequent	Response
83.3%	Aware of NBQA	66.7%	Aware of NBQA	62.1%	Aware of NBQA	84.2%	Aware of NBQA	84.2%	Aware of NBQA
25.0%	Nothing changed	26.7%	Contracts changed	16.7%	More efficient	25.0%	Nothing changed	40.0%	More efficient
20.0%	More efficient	21.4%	Purchasing more branded beef	16.7%	Contracts change	12.5%	Contracts changed	10.0%	Increased food safety
13.3%	Purchasing more value added	13.3%	Increased food safety	16.7%	Increased food safety	6.3%	Purchasing more branded beef	10.0%	Purchasing more branded beef
13.3%	Increased food safety	13.3%	More efficient			6.3%	Increased food safety		

^1^Government or trade organization

^2^Most frequent = top three most common responses and ties. Response data were evaluated as the number of times that interviewees in each market sector identified the attribute as a definition or description of the given category by the total number of responses for each market sector. When interviewees were unaware of the NBQA program, they were not asked what changes they had witnessed since the previous one occurred in 2016.

**Table 17.** Categorized responses from interviewed companies explaining changes witnessed since the 2016 National Market Cow and Bull Beef Quality Audit (NMCBBQ).

Packer (*n* = 13)		Retailer (*n* = 5)		Foodservice (*n* = 8)		Further processor (*n* = 7)		GTO^1^ (*n* = 35)	
Most frequent^2^	Response	Most frequent	Response	Most frequent	Response	Most frequent	Response	Most frequent	Response
76.9%	Aware of NMCBBQA	0.0%	Aware of NMCBBQA	0.0%	Aware of NMCBBQA	57.1%	Aware of NMCBBQA	54.3%	Aware of NMCBBQA
40.0%	Nothing					50.0%	Nothing	35.7%	Nothing
40.0%	Increased animal welfare					25.0%	Contracts changed	21.4%	Increased animal welfare
30.0%	Increased efficiency					25.0%	Specifications changed	14.3%	Increased efficiency
10.0%	Contracts changed							14.3%	Increased food safety initiatives

^1^Government or trade organization

^2^Most frequent = top three most common responses and ties. Response data were evaluated as the number of times that interviewees in each market sector identified the attribute as a definition or description of the given category by the total number of responses for each market sector. When interviewees were unaware of the NMCBBQA program, they were not asked what changes they had witnessed since the previous one occurred in 2016.

### Sustainability

**Figure 2.**
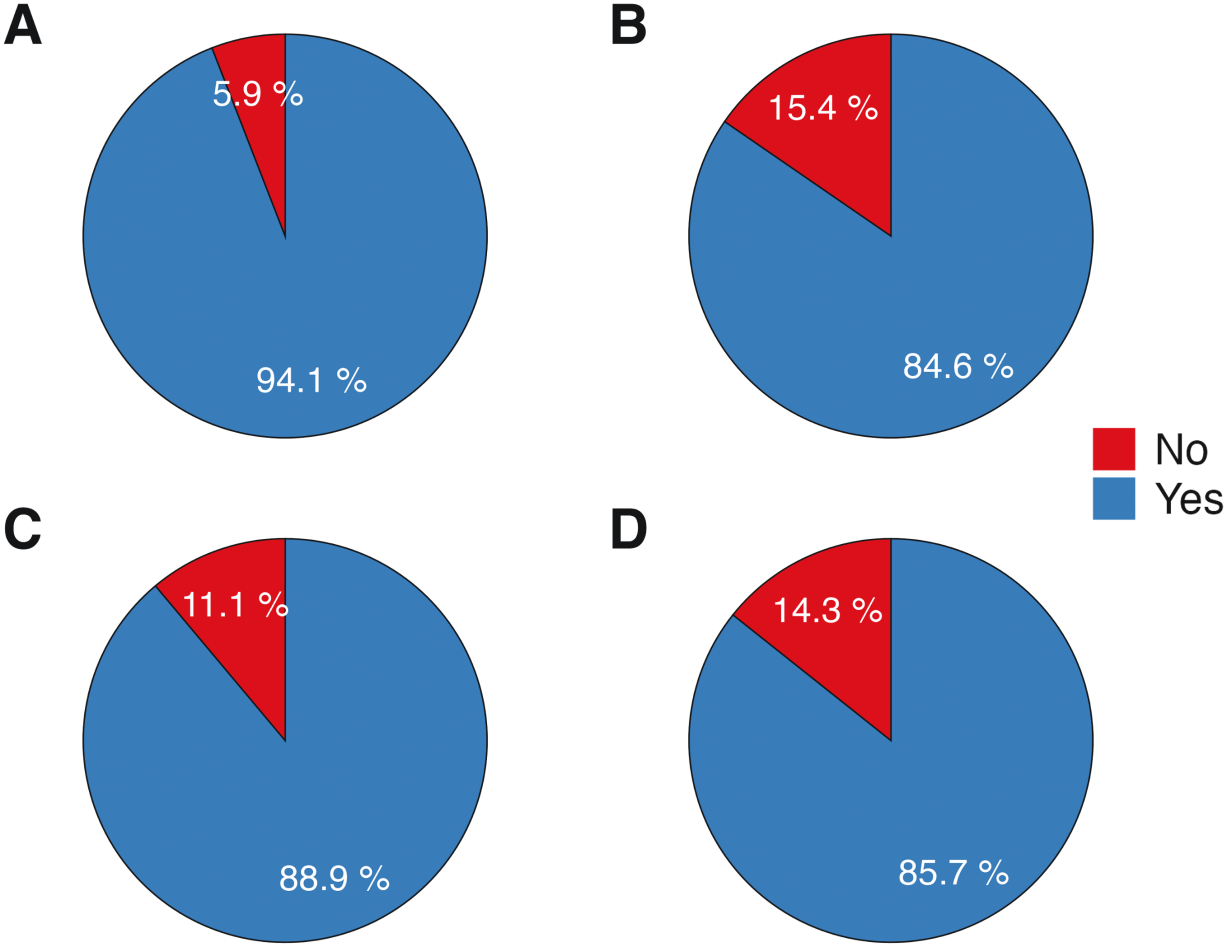
Percentage of each market sector for both the steer and heifer and market cow and bull beef markets: A) packers (*n* = 18), B) retailers (*n* = 14), C) foodservice (*n* = 11), D) further processor (*n* = 14) that indicated their company had sustainability goals.

Sustainability efforts are increasing across most industries, and many of these initiatives are being newly developed and implemented in companies worldwide (Hazaea et al., 2022). In an attempt to understand how the beef industry views and understands sustainability within their company, packers, retailers, foodservice, and further processors were asked three questions concerning their company’s sustainability goals and programs. Firstly, to the question “Does your company have sustainability goals?”, 94.1% of packers, 84.6% of retailers, 89.9% of foodservice, and 85.7% of further processors claimed their companies had such goals (Fig. 2). If a company claimed they had sustainability goals, the interview was logically routed to ask, “What are your company’s sustainability goals?” To this question, environmental goals were the most frequently reported, although some reported both social and economic goals of the scope 3 sustainability framework (Purvis et al., 2019). However, nearly one-third were unaware of what their company’s sustainability goals were or how they would be achieved (Table 18). Of note, these questions were asked to interviewees who are in positions to purchase beef and beef products, and therefore may not have been as familiar with their companies’ sustainability efforts as there was not a direct impact on their roles. However, it did not appear that sustainability efforts have been implemented within all functional working levels of companies. Despite the lack of workers’ knowledge of sustainability programming within their companies, it is reasonable to expect that in future iterations of NBQA, interviewees will become more familiar with sustainability measures their companies are making. Similar successes have been noted with top-down approaches such as food safety, as seen in the present study. Furthermore, a majority of those aware of their company’s sustainability goals reported that these goals involved the entire supply chain—not just their company (Table 19), in a scope 3 format for climate impact. Sustainability initiatives impacting the entire supply chain could have impacts on every market sector and far-upstream suppliers such as cow/calf and seedstock farms/ranches. Finally, respondents reported that they believed sustainability is a topic that will remain in the conversation for the next several years, and increased public interest will drive it to the forefront.

**Table 18.** Categorized responses from interviewed companies explaining their company’s sustainability goals for both the fed steer and heifer and market cow and bull beef markets.

Packer (*n* = 18)		Retailer (*n* = 14)		Foodservice (*n* = 11)		Further processor (*n* = 14)	
Most frequent^1^	Response	Most frequent	Response	Most frequent	Response	Most frequent	Response
64.7%	Environmental	58.3%	Environmental	67.7%	Environmental	61.5%	Environmental
23.5%	Unknown	33.3%	Unknown	33.3%	Unknown	30.8%	Unknown
23.5%	Social	25.0%	Social	0.0%	Social	23.1%	Social
11.8%	Economical	0.0%	Economical	0.0%	Economical	23.1%	Economical

^1^Most frequent = top three most common responses and ties. Response data were evaluated as the number of times that interviewees in each market sector identified the attribute as a definition or description of the given category by the total number of responses for each market sector.

**Table 19.** Categorized responses from interviewed companies explaining if their company’s sustainability goals are for their company or entail the entire beef supply chain.

Packer (*n* = 18)		Retailer (*n* = 14)		Foodservice (*n* = 11)		Further processor (*n* = 14)	
Most frequent^1^	Response	Most frequent	Response	Most frequent	Response	Most frequent	Response
58.8%	Entire supply chain	66.7%	Entire supply chain	55.6%	Entire supply chain	50.0%	Entire supply chain
29.4%	Owned operations	16.7%	Owned operations	11.1%	Owned operations	21.4%	Owned operations
11.8%	Unknown	16.7%	Unknown	16.7%	Unknown	14.3%	Unknown

^1^Most frequent = top three most common responses and ties. Response data were evaluated as the number of times that interviewees in each market sector identified the attribute as a definition or description of the given category by the total number of responses for each market sector.

### COVID-19 Pandemic

The COVID-19 pandemic affected nearly every facet of life for everyone in the United States. The beef industry was no exception. Since data for this study were collected during this tumultuous time, researchers felt it pertinent to include questions pertaining to the impacts of the pandemic on the beef industry and be able to use that information to help make sense of answers given in other portions of the interview. Across respondents, two main themes were commonly found in responses to the question, “What were the positive impacts of the COVID-19 pandemic on the beef industry?” First, sales of U.S. beef increased at retail and drove considerable demand for home cooking (at the expense of sales in foodservice). Second, the industry was forced to make rapid adaptations to keep up with demand in the wake of a reduced harvest capacity and workforce (Table 20). Conversely, struggles created by the pandemic also were discussed. Constituting the bulk of the negative impacts were labor shortages and supply chain failures (Table 21). Moreover, retailers, further processors, and foodservice operators frequently mentioned challenges with workmanship and finding products that met specifications. One retailer even said, “Negative [aspects of the COVID-19 pandemic] was and continues to be product availability and workmanship. However, we are willing to take just about anything at this point.” It is reasonable to assume, that most of the negatives mentioned by interviewees were the consequence of reduced harvest and fabrication capacity brought about by the pandemic and labor shortages.

**Table 20.** Categorized responses from interviewed companies explaining what they believed were the positive outcomes of the COVID-19 pandemic to the beef industry.

Packer (*n* = 24)		Retailer (*n* = 17)		Foodservice (*n* = 26)		Further processor (*n* = 18)		GTO^1^ (*n* = 42)	
Most frequent^2^	Response	Most frequent	Response	Most frequent	Response	Most frequent	Response	Most frequent	Response
60.9%	Beef performed^3^	56.3%	Beef performed	55.0%	Forced needed adaptations	62.5%	Forced needed adaptations	55.0%	Beef performed
34.8%	Forced needed adaptations	25.0%	Showed employer empathy	45.0%	Beef performed	50.0%	Beef performed	40.0%	Forced needed adaptations
26.1%	Showed employer empathy^4^	18.8%	Forced needed adaptations	30.0%	Beef industry’s resilience highlighted	25.0%	Revealed unknown weaknesses	22.5%	Revealed unknown weaknesses
17.4%	Beef industry’s resilience highlighted	12.5%	Revealed unknown weaknesses	20.0%	United the industry	18.8%	Beef industry’s resilience highlighted	15.0%	Showed employer empathy
17.4%	United the industry			15.0%	Showed employer empathy				

^1^Government or trade organization

^2^Most frequent = top three most common responses and ties. Response data were evaluated as the number of times that interviewees in each market sector identified the attribute as a definition or description of the given category by the total number of responses for each market sector.

^3^Beef performed = demand for beef products increased and were sought after during the COVID-19 pandemic.

^4^Showed employer empathy = companies increased well-being for workers.

**Table 21.** Categorized responses from interviewed companies explaining what they believed were the negative outcomes of the COVID-19 pandemic to the beef industry.

Packer (*n* = 24)		Retailer (*n* = 17)		Foodservice (*n* = 25)		Further processor (*n* = 18)		GTO^1^ (*n* = 42)	
Most frequent^2^	Response	Most frequent	Response	Most frequent	Response	Most frequent	Response	Most frequent	Response
61.9%	Labor struggles	58.8%	Supply chain malfunctions	56.5%	Labor Struggles	68.4%	Supply chain malfunctions	47.5%	Supply chain malfunctions
47.6%	Supply chain malfunctions	35.0%	Labor struggles	52.2%	Supply chain malfunctions	47.4%	Labor struggles	45.0%	Labor struggles
19.0%	Social concerns^3^	29.4%	Market volatility	30.4%	Market volatility	31.6%	Market volatility	32.5%	Social concerns

^1^Government or trade organization

^2^Most frequent = top three most common responses and ties. Response data were evaluated as the number of times that interviewees in each market sector identified the attribute as a definition or description of the given category by the total number of responses for each market sector.

^3^Social concerns = concerns about workers safety and social ramifications of continuing production operations amid the COVID-19 pandemic.

## Conclusions

The popularity of branded beef programs increased since 2016, with the average number of programs being sorted nearly doubling for each sector except for further processors. This could be a response to weaknesses identified in the NBQA-2016 where many sectors complained of “lacking marketing programs,” which have notably become a strength over the time elapsed between audits (Hasty et al., 2017). Furthermore, the industry has also increased the use of animal well-being programs.

“Food safety” was clearly most important for all market sectors, in both the fed steer and heifer and the market cow and bull beef markets. The importance of “food safety” was also reflected in characterizations where the most common answer was that food safety is “everything” and companies were not willing to pay a premium for food safety guarantees. Notably, producing safe products is paramount to companies’ success, and producing unsafe beef products could result in millions of dollars lost to civil liability, recalls, and brand reputation damage. Therefore, it was not surprising that food safety was also characterized as “a way of business.” Culture of “food safety” in the beef supply chain market sectors clearly has evolved and become significantly more pertinent in the beef industry since the last NBQA was conducted. Companies must produce safe beef to remain competitive in the market.

Additionally, “cattle genetics” and “eating satisfaction” were intertwined within the market sections for fed steer and heifer beef. Most likely caused by branded beef and marketing programs. Further, the data support that “cattle genetics” was the most likely quality factor to be a must-have across the market sectors. Furthermore, “eating satisfaction” is synonymous with “customer satisfaction,” and the market sectors associate “eating satisfaction” with high-quality cattle genetics to guarantee that customer satisfaction is maintained with high-quality beef products. In the beef industry, sustainability is viewed primarily as an environmental concept, and sustainability initiatives will continue to increase, impacting the entire supply chain. However, unlike food safety, sustainability initiatives have not reached a majority of the workforce.

Companies’ requirements to purchase beef were much less stringent compared to previous NBQAs, with the vast majority not willing to pay premiums to ensure a quality factor. Potentially, an impact of the COVID-19 pandemic. Moreover, the pandemic also created conditions that changed demand pressures, and the beef industry worked through these challenges while struggling with supply and labor shortages. Still yet, many recipients of beef products were unsatisfied with the workmanship and availability of beef products as a result of the shortages the pandemic created.

The fed steer and heifer industry data showed that the market sectors believe in American beef and stand behind the quality of the product. Additionally, among the market sectors, the image of the beef industry has improved since the last NBQA in 2016 for both the fed steer and heifer and market cow and bull beef markets. Although the image has improved, some struggles persist. Specifically in the market cow and bull beef market where concerns about animal well-being were more common. Despite criticisms, market sectors recognized the positive “secondary value” the market cow and bull beef market provides.
